# Grain and starch granule morphology in superior and inferior kernels of maize in response to nitrogen

**DOI:** 10.1038/s41598-018-23977-0

**Published:** 2018-04-20

**Authors:** Fucheng Zhao, Liquan Jing, Decheng Wang, Fei Bao, Weiping Lu, Guiyue Wang

**Affiliations:** 1 Dongyang Institute of Maize Research, Zhejiang Academy of Agricultural Sciences, Dongyang, Zhejiang 322100 China; 2 Key Laboratory of Crop Genetics and Physiology of Jiangsu Province, Yangzhou University, Yangzhou, Jiangsu 225009 China; 3 Jiangsu Hongqi Seed Co., Ltd., Taizhou, Jiangsu 225300 China

## Abstract

Maize (*Zea mays* L.) contributes approximately 55% of China’s grain production. The effects of nitrogen (N) on maize grain morphology and starch granules remain elusive. In this study, a field experiment in clay loam soil was conducted using three maize hybrids (Suyu 30, Suyu 20, and Suyu 29) and four N levels (0, 360, 450, and 540 kg ha^−1^) in 2010 and 2012. The results indicated that increased grain length and width, starch granule number, surface area, and volume, was associated with the application of 450 kg ha^−1^ of N. Differences between superior (ear base) and inferior (apical) grains decreased under highest yield treatments. The effects of N levels on inferior grains was more than that on superior grains. The starch granules of superior grains showed more polygonal, and bigger shape than inferior grains. The results revealed that N levels affected size and morphology of starch granules and grains. The application of 450 kg N ha^−1^ resulted in larger-sized starch granules and less difference between superior and inferior grains.

## Introduction

Maize has been associated with the largest plant area and highest total yield among various cereal crops, contributing to approximately 55% of China’s grain production. Similar to previous studies involving rice^1,2^ and wheat^3^, the later silk spikelets, which are usually located on the apical part of the ear, are either sterile or show poor grain filling activity^4^, thus rendering these as inferior. ‘Super’ rice, which possesses numerous spikelets in a panicle, are also unable to generate a high yield due to poor grain filling of its later-flowering inferior spikelets^2,5^. This problem involving inferior spikelets is further aggravated in the newly bred ‘super’ rice cultivars^6^. The maize grains in the lower and middle parts of the ear (superior grains) usually produce silk and fertilize early due to primigenic dominance^7^. However, the mechanism of grain filling in inferior and superior spikelets remains unknown and the relationship between grain filling in inferior spikelets and nitrogen (N) requirement remains elusive. It is therefore essential to investigate the physiological and ecological characteristics of superior and inferior grains and establish the mechanism of increased grain production.

Moisture regulates the length and surface area of grains, thus influencing grain yield^8^. It has been previously reported that panicles harboring smaller grain morphological features (length and width) significantly reduce rice yield in China^9^. In addition, it has been previously shown that shading, leaf cutting, spikelet removal, and increase in CO_2_ levels significantly improved the grain filling capacity of inferior grains^10^. It has been recently suggested that a metabolic interaction between polyamines and ethylene biosynthesis mediates grain filling in inferior rice spikelets^11^. Maize showed similar law. Shen *et al*.^12^ reported that apical kernel (inferior grains) of maize reacted differently to the nitrogen supply. That is, apical kernel developed well at an early grain filling stage and resulted in a higher kernel number, kernel weight and grain yield with better ear characteristics at maturity. Similar to response of rice starch^13^, Maize starch, whose morphology could be regulated by circumstances change (for example, high temperature)^14^, is stored as discrete semi-crystalline granules and consists of two main components, linear amylose and highly branched amylopectin^15^. Several studies have identified factors that influence the properties of starch granules, including long amylopectin branches, crystallinity, dense packing and restricted mobility of starch molecules, helix form, lamellar organization, and structural features of granules^16–23^. However, most of these studies mainly focused on the quality and physicochemical characteristics of starch, and information on the relationships between grain morphology and maize yield in superior and inferior grains is limited. Hence, this study was conducted to determine the relationship between yield and grain morphology in superior and inferior grains receiving various N levels and planted at a density of 82,500 plants ha^−1^.

## Results

### Effect of N rates on Grain Yield and Morphology

**Figure 1:**
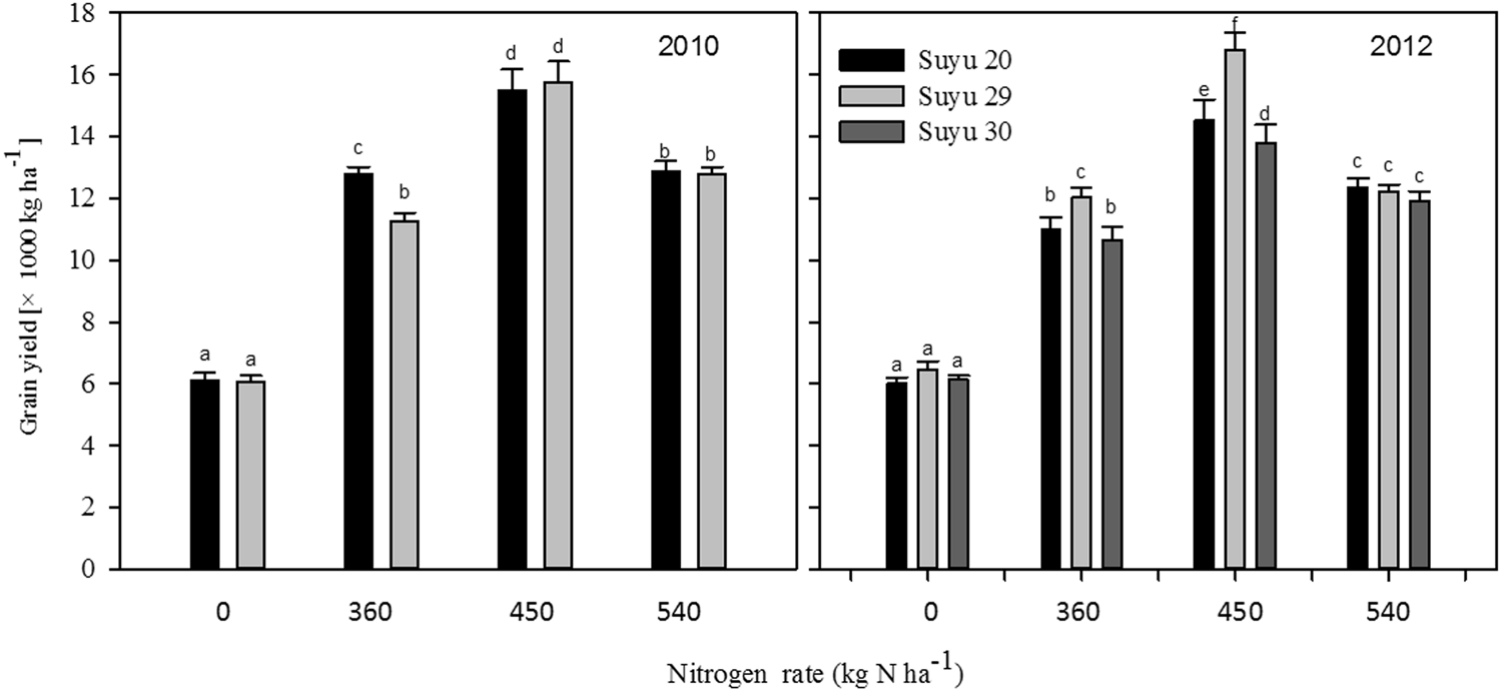
Effect of nitrogen levels on the grain yield of summer maize.

The application of various N levels resulted in a significant increase in yield of the three maize hybrids, Suyu 20, Suyu 29 and Suyu 30 (Fig. 1). As the N level increased, the yield of the three varieties presented single-peak curves, reaching peak values with 450 kg N ha^−1^ in both 2010 and 2012.

Tables 1 and 2 illustrate that that 450 kg N ha^−1^ markedly improved the morphological characteristics of superior and inferior grains (except grain thickness) compared to 0 kg N ha^−1^. At 450 kg N ha^−1^, the variation in the grain density and 1,000-grain weight in both superior and inferior grains was minimal. The variance values of grain density and 1,000-grain weight of the three hybrids (Suyu 20, Suyu 29 and Suyu 30, respectively; the same as follows) were 0.5, 2.1, and 15.3 g L^−1^ and 82.8, 33.5, and 50.3 g, respectively. ANOVA results showed that the effect of N levels on inferior grains was greater than that on superior grains for grain density and 1,000-grain weight. For example, compared to 0 kg N ha^−1^, the application of 450 kg N ha^−1^ resulted in an improved grain density and 1,000-grain weight in inferior grains from the three hybrids, which measured 2.3, 1.5, and 1.0 and 1.9, 1.9, and 2.1 times higher than those of superior grains, respectively. On average of four nitrogen levels, the higher geometric volume of superior and inferior grains of the three hybrids were 1.6, 1.7, and 1.8 and 1.4, 1.5, and 1.6 times higher than those of volume of grain, respectively, suggesting that sinking strength was still an important factor in generating a high-yield maize population. In terms of the F-value, no significant differences in the effect of N levels on grain thickness were detected for superior grains over inferior grains. And, significant Cultivar × N treatment interactions were detected for any parameters of morphology structure of maize grains except thickness of superior grains The effect of various N levels on inferior grains was generally higher than on superior grains and the application of 450 kg N ha^−1^ decreased the variation between superior and inferior grains and increased grain length and width, thus improving maize sink and yield.

**Table 1:** Effect of nitrogen levels on the morphology structure of superior grains.

Year	Hybrid	N Treatment	Grain density	1000-grain weight	geometrical morphology of grain (cm)			Geometric volume of grain	Volume of grain
		kg ha^−1^	(g L^−1^)	(g)	Length	Width	Thickness	(cm^3^)	(cm^3^)
2010	Suyu20	0	1109 ± 35a	312.5 ± 13.2a	0.89 ± 0.04a	0.81 ± 0.02a	0.49 ± 0.02a	0.35 ± 0.01a	0.28 ± 0.01a
		360	1150 ± 27bc	416.3 ± 14.5d	1.03 ± 0.02c	0.94 ± 0.02b	0.52 ± 0.01a	0.5 ± 0.01c	0.36 ± 0.00c
		450	1198 ± 26c	439.5 ± 9.3e	1.24 ± 0.03e	1.06 ± 0.01c	0.53 ± 0.02a	0.7 ± 0.02f	0.37 ± 0.00c
		540	1124 ± 31ab	422.4 ± 8.8de	1.11 ± 0.01d	0.93 ± 0.02b	0.53 ± 0.01a	0.55 ± 0.01d	0.38 ± 0.00c
	Suyu29	0	1101 ± 36a	305.3 ± 10.8a	0.94 ± 0.03b	0.83 ± 0.01a	0.52 ± 0.02a	0.41 ± 0.01b	0.28 ± 0.01a
		360	1170 ± 27bc	368.1 ± 14.9b	1.03 ± 0.01c	0.92 ± 0.02b	0.51 ± 0.02a	0.48 ± 0.01c	0.31 ± 0.01b
		450	1196 ± 29c	426.9 ± 7.7de	1.21 ± 0.02e	1.09 ± 0.01c	0.52 ± 0.01a	0.69 ± 0.02e	0.36 ± 0.00c
		540	1131 ± 26ab	376.2 ± 9.2c	1.03 ± 0.03c	0.91 ± 0.02b	0.52 ± 0.02a	0.49 ± 0.01c	0.33 ± 0.01bc
2012	Suyu30	0	1123 ± 26a	234.7 ± 9.1a	0.96 ± 0.01b	0.69 ± 0.01a	0.51 ± 0.02b	0.34 ± 0.02a	0.21 ± 0.00a
		360	1198 ± 37bc	278.7 ± 14.6b	1.06 ± 0.03cd	0.81 ± 0.02b	0.47 ± 0.01ab	0.4 ± 0.02c	0.23 ± 0.00b
		450	1222 ± 38c	299.4 ± 7.9d	1.1 ± 0.03d	0.91 ± 0.02cd	0.48 ± 0.02ab	0.48 ± 0.01e	0.24 ± 0.01bc
		540	1195 ± 34bc	281.2 ± 11.8bc	1.09 ± 0.01cd	0.87 ± 0.01c	0.45 ± 0.02a	0.43 ± 0.02d	0.24 ± 0.01b
	Suyu20	0	1113 ± 37a	264.5 ± 12.1d	0.92 ± 0.02a	0.85 ± 0.02bc	0.47 ± 0.02ab	0.37 ± 0.01b	0.24 ± 0.01d
		360	1152 ± 34b	392.4 ± 12.3f	1.02 ± 0.04c	0.96 ± 0.02d	0.53 ± 0.01b	0.52 ± 0.01f	0.34 ± 0.00f
		450	1192 ± 34bc	418.8 ± 11.0g	1.23 ± 0.03e	1.08 ± 0.03e	0.52 ± 0.02b	0.7 ± 0.01h	0.35 ± 0.01g
		540	1131 ± 38ab	390 ± 10.1f	1.16 ± 0.03e	0.92 ± 0.03cd	0.53 ± 0.02b	0.56 ± 0.02g	0.34 ± 0.01g
	Suyu29	0	1106 ± 32a	264.3 ± 14.9c	0.98 ± 0.04b	0.82 ± 0.02b	0.53 ± 0.02b	0.42 ± 0.01cd	0.24 ± 0.00c
		360	1172 ± 36bc	348.2 ± 9.2e	1.05 ± 0.04c	0.9 ± 0.01cd	0.51 ± 0.01b	0.48 ± 0.02e	0.3 ± 0.00e
		450	1183 ± 32bc	405.9 ± 9.2f	1.19 ± 0.03e	1.08 ± 0.01e	0.52 ± 0.01b	0.67 ± 0.01h	0.34 ± 0.01f
		540	1143 ± 39ab	353.7 ± 11.0e	1.05 ± 0.03c	0.93 ± 0.02cd	0.52 ± 0.02b	0.51 ± 0.02f	0.31 ± 0.01e
F-value		Year	0.06 NS	245.81**	10.19*	0.51NS	0.05NS	2.3NS	369.21NS
		Hybrid	11.72**	758.82**	13.19**	101.73**	5.2*	169.73**	1539.3**
		N treatment	45.76**	858.57**	550.48**	240.31**	0.16NS	492.42**	913.87**
		Year × Hybrid	0.01NS	1.97NS	0.01NS	3.16NS	0.15NS	0.52NS	1.02NS
		Year × N treatment	0.48NS	8.94*	5.78**	0.26NS	0.07NS	2.3NS	21.93**
		Cultivar × N treatment	1.3*	48.39**	35.23**	5.73**	2.22NS	21.6**	107.99**
		Year × Hybrid×N treatment	0.04NS	0.35NS	1.87NS	1.4NS	0.25NS	0.18NS	1.63NS

Means in the same column followed by the same letter do not differ statistically at the 0.05 probability level by an ANOVA protected Duncan’s test; *^,^**NS Significantly different at the 0.05 and 0. 01 probability levels and no significant difference, respectively.

**Table 2:** Effect of nitrogen levels on the morphology structure of inferior grains.

Year	Hybrid	N Treatment	Grain density	1000-grain weight	geometrical morphology of grain (cm)			Geometric volume of grain	Volume of grain
		kg ha^−1^	(g L^−1^)	(g)	Length	Width	Thickness	(cm^3^)	(cm^3^)
2010	Suyu20	0	1013 ± 39a	180.3 ± 10.6a	0.72 ± 0.01a	0.76 ± 0.03a	0.51 ± 0.02a	0.28 ± 0.01a	0.18 ± 0.00a
		360	1065 ± 39c	286.4 ± 10.7c	1.01 ± 0.01d	0.82 ± 0.01b	0.52 ± 0.02ab	0.43 ± 0.01d	0.27 ± 0.01c
		450	1196 ± 27d	328.7 ± 13.3e	1.12 ± 0.03e	0.94 ± 0.02c	0.53 ± 0.01ab	0.56 ± 0.02e	0.27 ± 0.00c
		540	1089 ± 36c	302.3 ± 8.5d	1.03 ± 0.03d	0.77 ± 0.02a	0.54 ± 0.02b	0.42 ± 0.01d	0.28 ± 0.01cd
	Suyu29	0	1060 ± 37bc	212.4 ± 13.7b	0.84 ± 0.01b	0.74 ± 0.02a	0.51 ± 0.02a	0.32 ± 0.01b	0.2 ± 0.01b
		360	1121 ± 326c	328.7 ± 8.8e	1.01 ± 0.02d	0.82 ± 0.02b	0.51 ± 0.01a	0.43 ± 0.01d	0.29 ± 0.01d
		450	1190 ± 38d	403.6 ± 14.5f	1.1 ± 0.02e	0.94 ± 0.01c	0.52 ± 0.02ab	0.54 ± 0.01e	0.34 ± 0.00e
		540	1104 ± 38c	329.7 ± 14.8e	0.93 ± 0.01c	0.77 ± 0.03a	0.51 ± 0.01a	0.37 ± 0.02c	0.3 ± 0.01d
2012	Suyu30	0	1110 ± 302b	158.9 ± 3.7a	0.68 ± 0.01a	0.68 ± 0.01a	0.39 ± 0.01a	0.18 ± 0.01a	0.14 ± 0.00a
		360	1146 ± 40bc	194.4 ± 3.1c	0.93 ± 0.02d	0.71 ± 0.01ab	0.48 ± 0.01bc	0.31 ± 0.02c	0.17 ± 0.01c
		450	1207 ± 37c	249.1 ± 4.3e	1.03 ± 0.02ef	0.76 ± 0.03c	0.47 ± 0.02bc	0.37 ± 0.01d	0.21 ± 0.01e
		540	1134 ± 26bc	215.5 ± 5.7b	0.9 ± 0.01d	0.71 ± 0.01ab	0.47 ± 0.02bc	0.3 ± 0.02c	0.19 ± 0.01b
	Suyu20	0	1023 ± 31a	181.8 ± 3.2b	0.75 ± 0.02b	0.75 ± 0.03bc	0.5 ± 0.01c	0.28 ± 0.01b	0.18 ± 0.00d
		360	1072 ± 38b	324.5 ± 6.4f	1.02 ± 0.03e	0.85 ± 0.03e	0.51 ± 0.01cd	0.45 ± 0.01f	0.3 ± 0.01f
		450	1193 ± 37c	364.1 ± 6.5h	1.1 ± 0.02f	0.93 ± 0.01f	0.55 ± 0.01d	0.56 ± 0.01h	0.31 ± 0.00f
		540	1089 ± 27b	324.2 ± 7.9g	1.06 ± 0.01ef	0.79 ± 0.02cd	0.54 ± 0.01d	0.45 ± 0.01f	0.3 ± 0.01g
	Suyu29	0	1066 ± 33b	198.3 ± 5.3d	0.86 ± 0.02c	0.75 ± 0.01bc	0.5 ± 0.01c	0.32 ± 0.01c	0.19 ± 0.01e
		360	1117 ± 38b	288 ± 6.3h	1.01 ± 0.02e	0.81 ± 0.02de	0.49 ± 0.02bc	0.4 ± 0.02e	0.26 ± 0.00h
		450	1185 ± 35c	362.1 ± 7.7i	1.07 ± 0.03f	0.94 ± 0.01f	0.51 ± 0.02cd	0.52 ± 0.01g	0.31 ± 0.01i
		540	1107 ± 28b	302.7 ± 4.6h	0.92 ± 0.01d	0.78 ± 0.01cd	0.5 ± 0.02c	0.36 ± 0.01d	0.27 ± 0.01h
F-value		Year	0.11NS	3.26NS	0.21NS	0.42NS	0.65NS	0.19NS	8.44**
		Hybrid	35.69**	837.03**	126.73**	77.93**	13.67**	241.16**	1486.75**
		N treatment	145.38**	1422.6**	1019**	111.23**	2.91*	461.9*	1551.04*
		Year × Hybrid	0.1NS	229.43**	4.35*	0.28NS	0.52NS	7.24*	338.44**
		Year × N treatment	0.23NS	0.35NS	5.05**	0.53NS	0.14NS	1.33NS	1.22NS
		Cultivar × N treatment	4.66*	48.93**	58.8**	4.54**	1.46**	14.86**	78.88**
		Year × Hybrid × N treatment	0.07NS	16.67**	1.00NS	0.87NS	0.13NS	0.67NS	23.99**

Means in the same column followed by the same letter do not differ statistically at the 0.05 probability level by an ANOVA protected Duncan’s test; *^,^**NS Significantly different at the 0.05 and 0. 01 probability levels and no significant difference, respectively.

### Effect of N on Starch Granules of Superior and Inferior Grains

**Figure 2:**
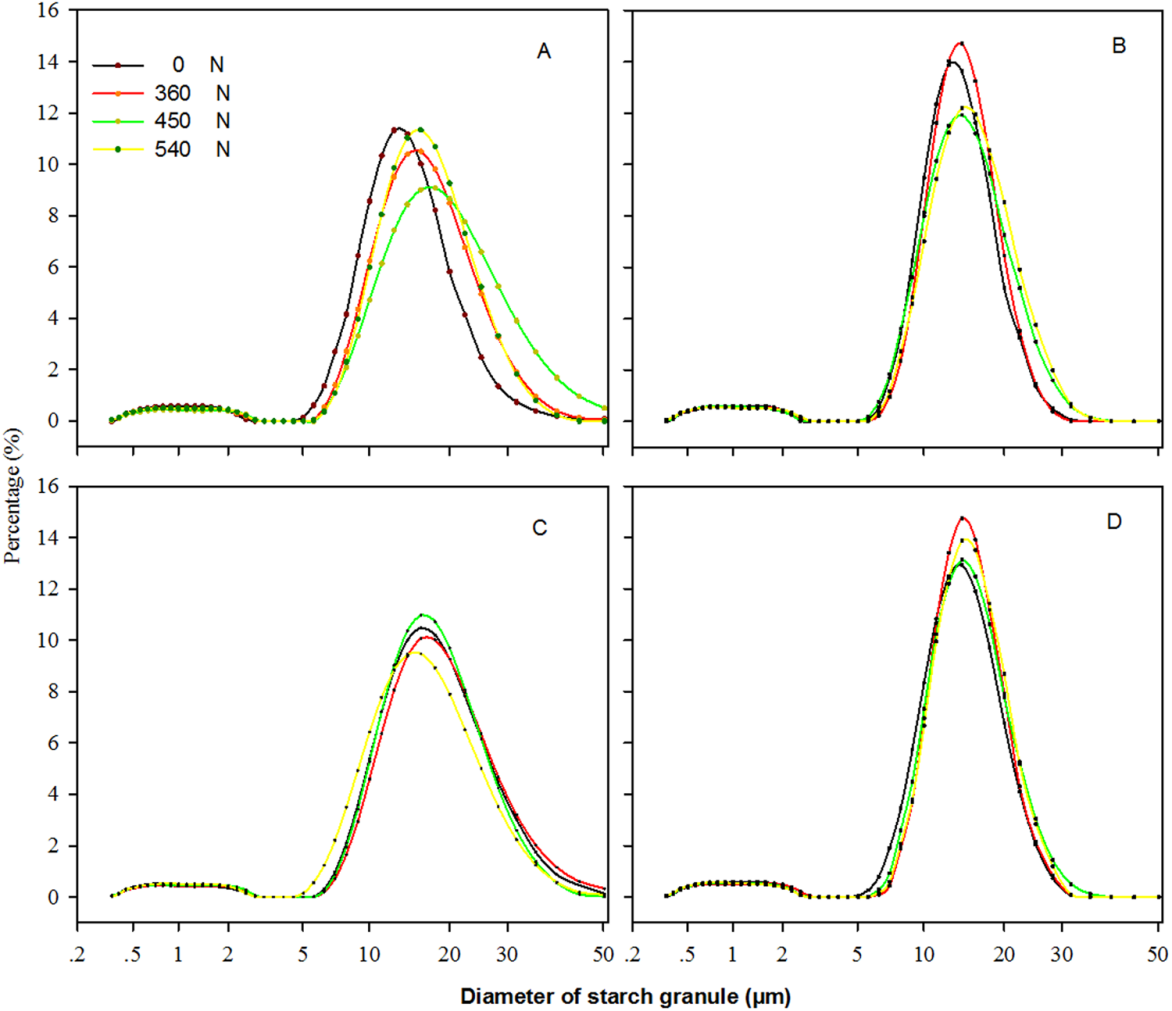
Effects of nitrogen levels on starch granule volume distribution of superior (**A** and **C**) and inferior (**B** and **D**) grains from Suyu 20 (**A**, and **B**), and Suyu 29 (**C** and **D**) in 2010.

**Figure 3:**
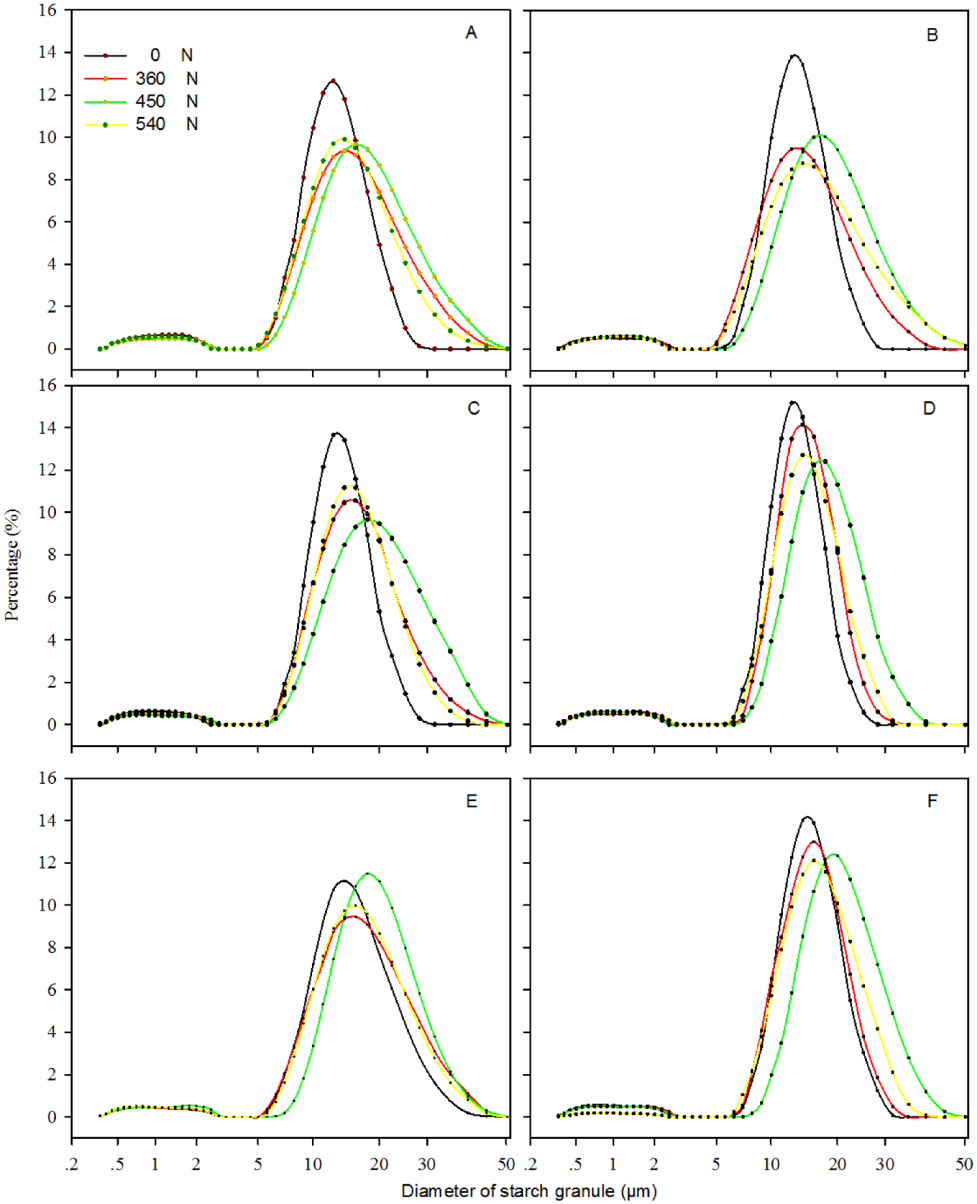
Effects of nitrogen levels on starch granule volume distribution of superior (**A**,**C** and **E**) and inferior (**B**,**D** and **F**) grains from Suyu 30 (**A** and **B**), Suyu 20 (**C** and **D**), and Suyu 29 (**C** and **D**) in 2012.

Figure 2 and Table 3 show the effects of N treatments on starch granules in superior and inferior grains. The effects on big starch granules were much greater than those on small starch granules. No significant differences in the diameter of starch granules <5 μm were observed among superior and inferior grains. The application of 450 kg N ha^−1^ resulted in a marked increase in the number of big starch granules. In the superior grains of Suyu 29, for example, the percentages starch granules with a diameter >15 μm using 0, 360, 450, and 540 kg N ha^−1^ were 49.13%, 57.93%, 64.16%, and 53.20% (Figs 2C and D and 3E and F). Figures 2 and 3 illustrate that the distribution of starch granules of superior grains was different from that of inferior grains; the starch granules of superior grains showed smaller peak diameters, but a wider distribution than that in inferior grains. In terms of F-value (Table 3), various N levels imparted significant differences in surface area and volume between superior and inferior grains from hybrids. And, significant hybrid × N treatment interactions were detected for surface area and volume of superior and inferior grains of maize. Overall, the application of 450 kg N ha^−1^ increased surface area and volume of starch granules, also, increased the number of big starch granules Furthermore, big starch granules improved maize yield compared to small starch granules. This may explain the observed improvement in grain density and 1,000-grain weight, which resulted in a higher maize yield.

**Table 3:** Effects of nitrogen levels on weighted average of surface area and volume of superior and inferior grains of maize.

Year	Hybrid	N Treatment	Surface area (μm^2^)		Volume (μm^3^)	
		kg ha^−1^	Superior	Inferior	Superior	Inferior
2010	Suyu20	0	6.45 ± 0.12g	6.34 ± 0.12i	17.5 ± 0.13lm	12.68 ± 0.49n
		360	7.68 ± 0.13cde	7.02 ± 0.10cde	19.36 ± 0.10i	14.43 ± 0.29j
		450	8.46 ± 0.11a	6.65 ± 0.06fghi	24.74 ± 0.17c	15.85 ± 0.37cd
		540	6.08 ± 0.10h	6.41 ± 0.06hi	16.56 ± 0.13n	15.38 ± 0.12f
	Suyu29	0	7.78 ± 0.12cde	7.64 ± 0.09a	20.42 ± 0.39g	15.68 ± 0.14e
		360	7.45 ± 0.10e	6.57 ± 0.13ghi	17.36 ± 0.11m	14.39 ± 0.08j
		450	8.57 ± 0.15a	7.15 ± 0.11bcd	25.15 ± 0.09b	15.91 ± 0.09c
		540	7.55 ± 0.1de	7.27 ± 0.09bc	16.54 ± 0.12n	15.26 ± 0.07g
2012	Suyu30	0	6.99 ± 0.20f	6.44 ± 0.23ghi	14.96 ± 0.28o	12.75 ± 0.35n
		360	7.48 ± 0.12e	7.28 ± 0.18bc	20.12 ± 0.24h	16.71 ± 0.27b
		450	7.83 ± 0.29cd	6.74 ± 0.17efg	23.74 ± 0.37d	14.46 ± 0.35j
		540	7.04 ± 0.26f	7.01 ± 0.22cde	18.37 ± 0.32k	17.54 ± 0.29a
	Suyu20	0	6.50 ± 0.20g	6.63 ± 0.2fghi	16.5 ± 0.29n	13.15 ± 0.25m
		360	7.71 ± 0.15cde	6.90 ± 0.23def	19.42 ± 0.26i	14.25 ± 0.36k
		450	8.28 ± 0.14ab	6.66 ± 0.15fgh	22.35 ± 0.29e	13.99 ± 0.16l
		540	7.07 ± 0.19f	6.75 ± 0.17efg	14.84 ± 0.3o	14.8 ± 0.42i
	Suyu29	0	7.96 ± 0.13bc	6.9 ± 0.28def	19.14 ± 0.23j	15.17 ± 0.32gh
		360	7.75 ± 0.15cde	7.08 ± 0.26cd	22.19 ± 0.27f	15.11 ± 0.24h
		450	8.35 ± 0.17a	7.18 ± 0.27bcd	26.92 ± 0.41a	15.84 ± 0.41cd
		540	7.48 ± 0.14e	7.42 ± 0.22ab	17.58 ± 0.38l	15.79 ± 0.28de
F-value		R2	0.684**	0.753**	0.997**	0.979**
		Year	1.24NS	1.15NS	8.14NS	12.91NS
		Hybrid	13.52**	38.76**	502.52*	269.54*
		N treatment	29.13**	30.61**	4721.69*	483.25*
		Year × Hybrid	0.51NS	1.65NS	621.02**	47.74**
		Year × N treatment	1.44NS	7.09**	27.79**	42.29**
		Cultivar × N treatment	3.04*	3.84**	121.08**	122.38**
		Year × Hybrid × N treatment	1.71NS	1.03NS	19.31**	21.74**

Means in the same column followed by the same letter do not differ statistically at the 0.05 probability level by an ANOVA protected Duncan’s test; *^,^**NS Significantly different at the 0.05 and 0. 01 probability levels and no significant difference, respectively.

### Correlation analysis

Table 4 illustrate that grain yield of maize positively and significantly correlated with any parameters of grain and starch granule of grain except surface area and volume of inferior grains. Compared with 1,000-grain weight of superior grains, there were more significant correlation relations with inferior grains. Based on the correlation coefficients, maize yield was most closely related to any morphology parameters of both superior and inferior grains, followed by 1000-grain weight and grain density.

**Table 4:** Correlations analysis among maize yield and the parameters of grain and starch granule of superior and inferior grain.

Grain morphology	Yield	S-Grain density	S-1000-grain weight	S-Length	S-Width	S-Thickness	S-geometric volume of grain	S-volume of grain	S-Surface area	S-Volume	I-Grain density	I-1000-grain weight	I-Length	I-Width	I-Thickness	I-geometric volume of grain	I-volume of grain	I-Surface area
S-Grain density	0.77**	1																
S-1000-grain weight	0.76**	0.29	1															
S-Length	0.90**	0.71**	0.69**	1														
S-Width	0.85**	0.55*	0.86**	0.82**	1													
S-Thickness	0.19	−0.28	0.63**	0.28	0.4	1												
S-geometric volume of grain	0.85**	0.53*	0.86**	0.92**	0.96**	0.52*	1											
S-volume of grain	0.65**	0.12	0.98**	0.59**	0.79**	0.69**	0.80**	1										
S-Surface area	0.51*	0.55**	0.38	0.56**	0.62**	0.35	0.64**	0.28	1									
S-Volume	0.54*	0.68**	0.38	0.59**	0.68**	0.09	0.64**	0.27	0.80**	1								
I-Grain density	0.77**	0.88**	0.35	0.83**	0.60**	0.01	0.68**	0.2	0.67**	0.71**	1							
I-1000-grain weight	0.82**	0.43	0.91**	0.76**	0.90**	0.57**	0.90**	0.86**	0.55*	0.45*	0.53*	1						
I-Length	0.90**	0.65**	0.83**	0.87**	0.86**	0.41	0.88**	0.74**	0.58**	0.58**	0.67**	0.87**	1					
I-Width	0.69**	0.45*	0.82**	0.72**	0.94**	0.50*	0.91**	0.75**	0.69**	0.71**	0.53*	0.84**	0.76**	1				
I-Thickness	0.45*	0	0.76**	0.42	0.69**	0.48*	0.62**	0.79**	0.22	0.24	0	0.64**	0.61**	0.64**	1			
I-geometric volume of grain	0.82**	0.51*	0.91**	0.84**	0.96**	0.53*	0.96**	0.84**	0.63**	0.63**	0.57**	0.91**	0.92**	0.93**	0.74**	1		
I-volume of grain	0.74**	0.29	0.92**	0.66**	0.85**	0.62**	0.83**	0.89**	0.45*	0.33	0.37	0.98**	0.82**	0.79**	0.69**	0.87**	1	
I-Surface area	0.07	0.08	0.01	0.03	0.12	0.05	0.07	0.01	0.46*	0.29	0.11	0.15	0.13	0.02	0.06	0.05	0.15	1
I-Volume	0.4	0.44*	0.17	0.46*	0.29	−0.1	0.31	0.13	0.34	0.36	0.43	0.25	0.42	0.09	0.14	0.25	0.2	0.63**

S: Super grain; I: Inferior grain; *^,^**Correlations are significant at the 0.05 and 0.01 levels, respectively.

### Starch Granule Morphology of Superior and Inferior Grains

**Figure 4:**
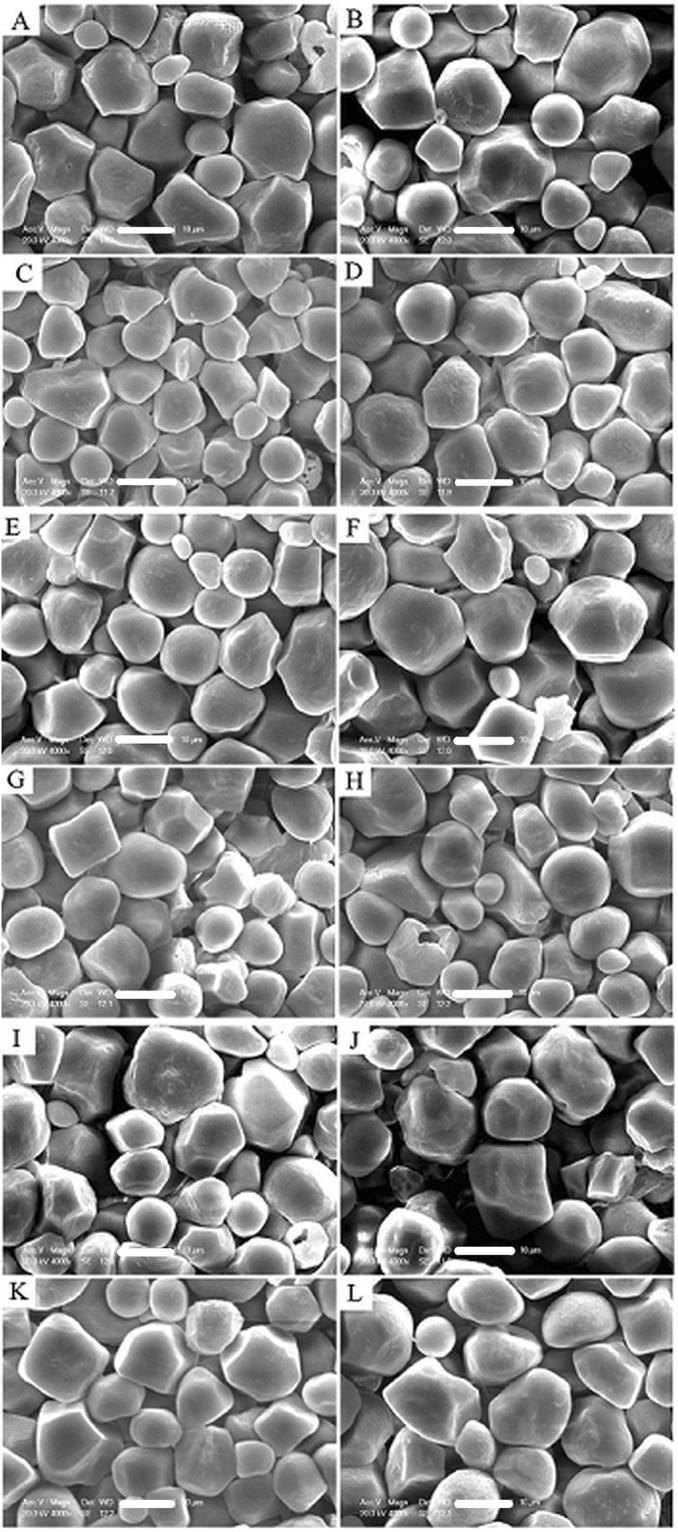
Scanning electron microscopy of the starch granules arrangement of superior(**A**,**B**,**E**,**F**,**I** and **J**) and inferior (**C**,**D**,**G**,**H**,**K**, and **L**) grains from Suyu 30(**A**–**D**), Suyu 20 (**E**–**H**) and Suyu 29 (**I**–**L**) and effected by 0 (**B**,**D**,**F**,**H**,**J** and **L**) and 450 (**A**,**C**,**E**,**G**,**I** and **K**) kg N ha^−1^ (4000×, Scale bar = 10 μm).

**Figure 5:**
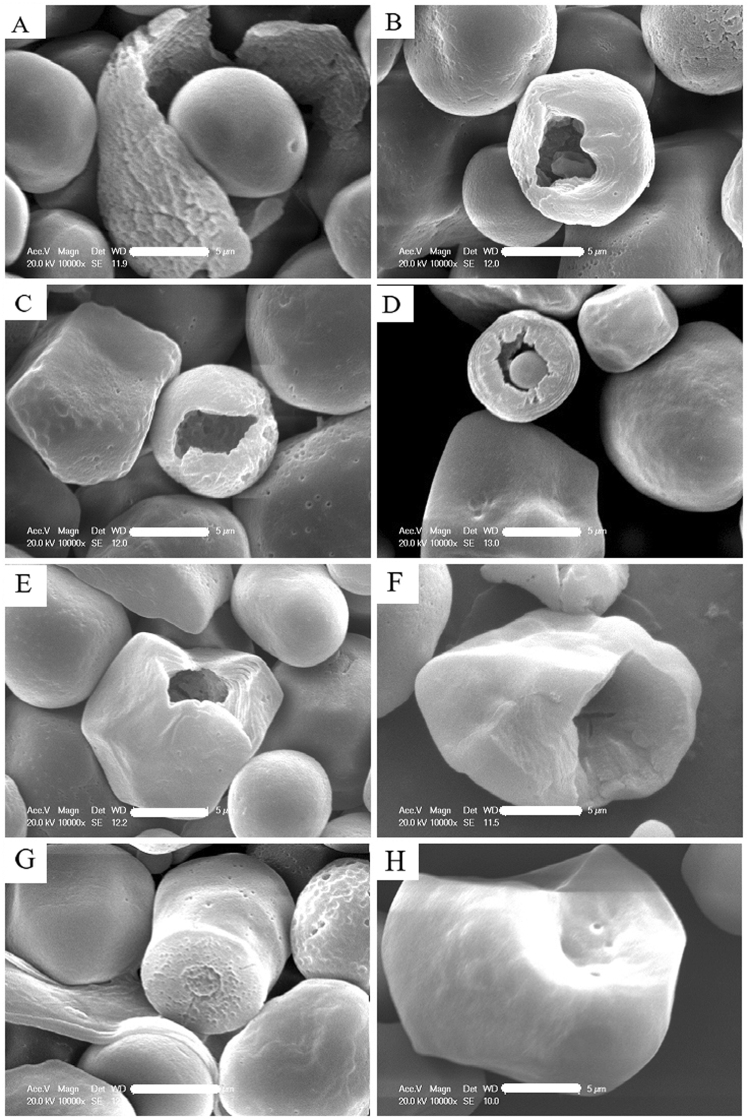
Scanning electron microscopy of the starch granules from mechanical damage of Suyu 30 (**A**,**C**, and **F**), Suyu 20 (**B**,**D** and **G**) and Suyu 29 (**E** and **H**) (10000×, Scale bar = 5 μm.).

The starch granule morphology of the three maize hybrids using 0 and 450 kg N ha^−1^ was examined using SEM. The granules showed polygonal and spherical shapes (Fig. 4), similar to the finding of Wei *et al*.^15^ using rice starch. However, several striking differences were observed between the superior and inferior grains. For example, the polygonal starch granules of superior grains (Fig. 4A,B,E,F,I and J) were bigger than inferior grains (Fig. 4C,D,G,H,K and L). Conversely, at 0 kg N ha^−1^, isolated starch granules from inferior grains with more smooth surfaces were spherical in shape (Fig. 4C,G and K). The SEM figures (10,000×) of the starch granules also showed mechanical damage (Fig. 5), ‘Hollow’ and ‘solid’ inner structures were also observed in the starch granules. The wall of spherical starch granules was thin and its chambers were large. The walls of the polyhedral starch granules were thick and layered and its chambers were small. These features (except starch granule damage) may explain the observed improvement in grain density and 1,000-grain weight, which improved maize production grain density.

## Discussion

### Grain and Starch Granule Morphology and its Relationship with Maize Yield

Improved sink activity, sources, and assimilate and nutrient flow are essential in increasing yield. Increasing the grain number currently serves as the main approach in improving sink activity in maize, although evidence on the relationship between morphology structure of grain and yield is limited. Previous studies have shown that grain morphology (length, width, and surface area) could be regulated by moisture, as evidenced by studies using rice^9^ or starch granule morphology of rice could be regulate by N level^13^. In cereals, maximum seed volume is established before reaching its maximum dry weight and this is generally estimated as the developmental stage when the kernel maximum water content is achieved^24^. The 1,000-grain weight of maize and length of grain are quantitative traits that are controlled by multiple genes and are often influenced by environmental factors^25^. Because the ovaries at the tip of the ear have not reached their maximum size at the silking stage^7^, the apical kernels of maize ears have smaller size and dry matter accumulation rates than basal kernels. However, this difference could be regulated by changing the temperature during growth^26^ and N levels^12^. Our research indicated that under high-density conditions (82,500 plants ha^−1^), the application of 450 kg N ha^−1^ also mitigated this difference in grain density and 1,000-grain weight between superior and inferior grains and significantly increased grain density, length, width, and volume of grains, with minimal effects on grain thickness. It appears that smaller differences in superior and inferior grains improved maize yield. N largely influenced grain morphology in inferior grains than that in superior grains. Our this result is very similar to one of previous reports^12^.

The starch content was positively correlated with the maize starch granule volume ranges of 0.8 to 2 μm, 2 to 10 μm, and 10 to 15 μm, but negatively correlated with other size ranges^18^. Starch granules with a diameter of ≥15 μm were predominant^19^. Temperature could also regulate starch granule size. Interestingly, temperature affected granule size, pasting temperature, and transition temperature, in which a lower temperature prevailed during the filling stage, resulting in larger starch granules and lower pasting and transition temperatures^20^. Our results demonstrated a positive correlation between large starch granules and maize yield. Furthermore, the predominance of large starch granules could be strongly influenced by N levels, although several striking differences between superior and inferior grains have been observed. For example, superior grains showed a wider distribution than inferior grains. In the superior grains of Suyu 29, the percentages of starch granules with a diameter of >15 μm using 0, 360, 450, and 540 kg N ha^−1^ were 49.13%, 57.93%, 64.16%, and 53.20% (Figs 2C and D and 3E and F) respectively.

### The Microstructure of Starch Granules

Recent developments in microscopy have allowed the examination of starch granule microstructures, which provide information on the physicochemical characteristics of maize starch, as well as grain filling mechanisms and yield. Most of the previous studies on the microstructure of starch granules mainly focused on three aspects of starch granules: internal structures, external structures, and physicochemical characteristics. A semi-compound granule generally contains one exterior surface and two or more hila, as described by Wei *et al*.^15^ and Nordmark *et al*.^21^. However, some starch granules have small cavities with a diameter range of 0.07 to 0.10 μm, as well as channels and pores randomly distributed across their surfaces, often in clusters and showing variations^22,23^. Evidences from SEM and chemical analyses have shown that the pores are randomly distributed, varying in number per granule, and the channels were irregularly bent^27^. These granules provide the architecture of blocklets, and a normal blocklet is mainly formed by crystalline and amorphous lamellae^28,29^ previously reported that the semi-crystalline blocklets generally consist of two types, ‘normal’ and ‘defective’, which occur within the same starch molecule and are the basic units that construct starch granules. The normal blocklets comprise the hard shells, whereas soft shells consist of the defective ones; both hard shells and soft shells are discontinuous structures^30^. Nordmark and Ziegler^21^ observed radially oriented crystalline lamellae using atomic force microscopy (AFM) and observed linear or lightly branched starch polymers composed of spherulites. Amylopectin plays a more important role in blocklet architecture, whereas the other component provides strength and flexibility to starch granules^31^. The length of the side chain of amylopectin varies with the size, as well as the inner and surface layers of starch granules^32,33^. We observed ‘hollow’ and ‘solid’ inner structures in the starch granules (Fig. 5); the wall of the spherical starch granules was thin and the chamber was big. The wall of the polyhedral starch granules was thick and layered and the chamber was small. Li *et al*.^29^ suggested that the hydrolysis of *α-*amylase or other chemicals that flowed into starch granules along channels were responsible for this ‘hollow’ formation. However, Wei *et al*.^13^ and our current study suspect that the physicochemical characteristics of granule walls, as well as the ‘hollow’ and ‘solid’ inner structures of starch granules, may generally occur in nature and are controlled by the conditions of the environment (e.g., N and temperature) during the filling stage, thus affecting maize yield. More extensive research on this area is therefore warranted.

## Materials and Methods

### Plant Materials and Growth Conditions

The experiment was conducted from June to October in 2010 and 2012 in clay loam soil plots at the Key Laboratory of Crop Genetics and Physiology of Yangzhou University (119°26′E, 32°24′N), Jiangsu Province, China. This site generally experiences a sub-tropical humid, monsoon climate. Three maize hybrids were analyzed, Suyu 20, Suyu 29, and Suyu 30. The first two hybrids have been extensively used in recent years in China, whereas Suyu 30 was authorized for propagation by the National Crop Variety Examination and Approving Committee in 2011, based on its high heterocyst and yield potential, especially in southeast China. The field of the test plots showed similar chemical property, agronomic histories and favorable conditions for irrigation and drainage Soil from a nearby clay loam field (0 to 20 cm depth) was collected, air-dried, and sieved through a 5-mm screen. The soil contained 96.71 mg kg^−1^ N (sum of NO_3−_ and NH_4_^+^-N), 33.3 mg kg^−1^ phosphorus (P), 129 mg kg^−1^ potassium (K), 28.5 g kg^−1^ organic matter, and 1.80 g kg^−1^ total N at pH 6.81.

### Experimental Design

**Figure 6:**
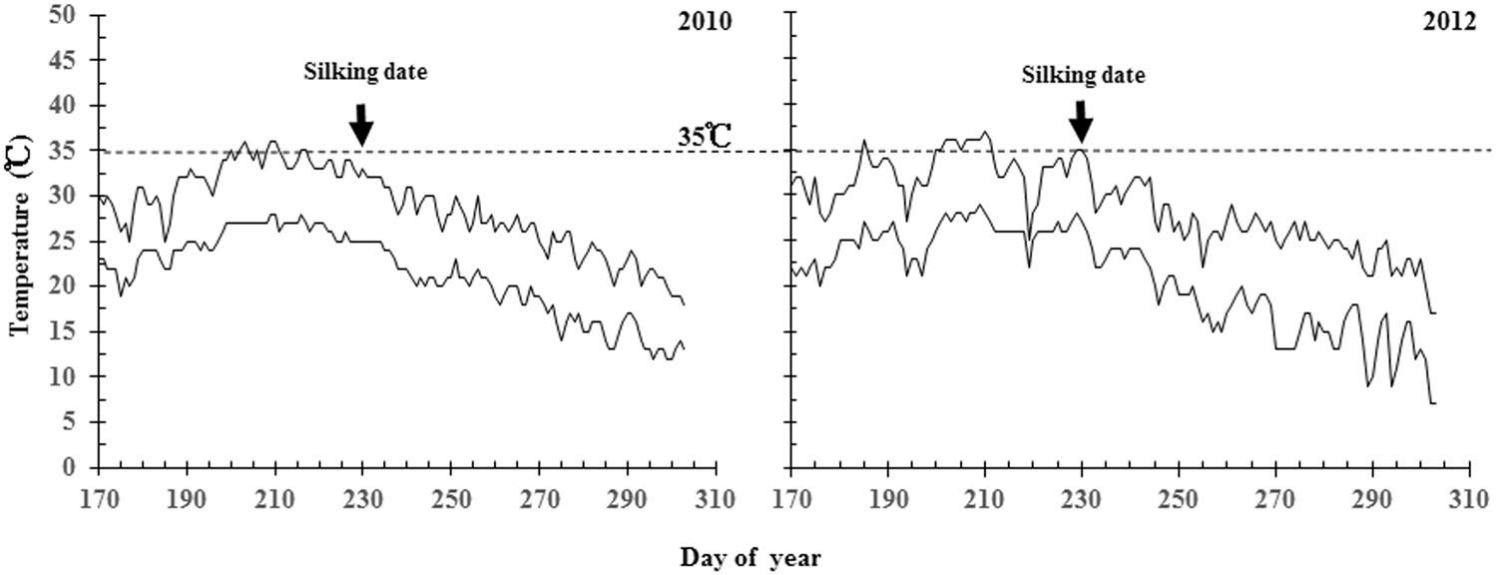
Temperature condition during the period from sowing to final harvest for the 2010 and 2012 growing seasons of maize. The minimum and maximum temperature are given for each day. Arrows indicate the start of silking date.

A split-plot experimental design consisting of three replicates was used in the study, with hybrids planted in the main plots and N treatments conducted in the sub-plots. Seeds were sowed on June 27 each year at a density of 82,500 plants ha^−1^ in test plot area of 110 m^2^. Planting followed a 0.3 m by 0.7 m row spacing pattern. Four N levels (Granule urea, N concentration = 46%) were utilized (0, 360, 450, and 540 kg ha^−1^) based on the previous experimental results in which the yield of maize reached its peak at 450 kg N ha^−1^. Vegetative and reproductive N treatments (Granule urea, N concentration = 46%) was incorporated to the soil at the 10-leaf and anthesis stages. Table 5 shows the N application scheme in detail. Base fertilizers of 150 kg ha^−1^ P_2_O_5_ and 225 kg ha^−1^ K_2_O were applied once upon planting. Figure 6 shows the temperature condition during the period from sowing to final harvest for the 2010 and 2012 growing seasons. Other field management measurements were carried out according to the maize requirements for high yield.

**Table 5:** Nitrogen fertilization scheme (kg ha^−1^).

N levels	Base-fertilizer	Ear-fertilizer	Grain- fertilizer
0	0	0	0
360	135	225	0
450	135	270	45
540	135	315	90

### Grain Morphology

Ten ears from each treatment were harvested 50 days after anthesis and fully dried. The grains of four rows at the base of the ear were removed and divided into 4 equal portions. The grains from the lower one-fourth portion were designated as superior, whereas those from the top one-fourth portion were defined as inferior. The length (a), width (b), thickness (c), and 1,000-grain weight of the superior and inferior grains were measured. The volume of the grains (approximately 100 g) was assessed using the 95% alcohol displacement method. The following calculations were performed: geometric volume of the grain = a × b × c; and grain density = weight of the grain (approximately 100 g)/volume of the grains.

### Isolation of Starch Granules

Starch granules were isolated according to the method of Wei *et al*.^15^ and Lu^35^, with minor modifications. The maize grains were steeped in 0.2% NaOH for 48 h at room temperature. The samples were then rinsed in ultrapure water and then ground using a blender for 3 min. The suspensions were filtered through a 100-mesh sieve. The materials collected on the screen were again homogenized for 2 min, and then passed through the same sieve. The filtrates were adjusted to a pH level of 9.5 with 0.2% NaOH and a pH controller, mixed with excess alkaline protease (Enzyme Commission (EC) Number 3.4.21.62, Beijing Solarbio Science & Technology Co., Ltd., China), and stirred at 42 °C the pH was adjusted when it dropped below pH 8.5) . Approximately 18 h later, the starch was washed with 0.2% NaOH and centrifuged (4,000 *g* for 10 min) until no biuret reaction occurred (or protein content was less than 0.5%), and finally washed with water. The starch was further treated with anhydrous ethanol and a mixture of methanol and chloroform (v/v = 1:1) three times. Finally, the starch was collected and dried under atmosphere, ground into powder, and then passed through a 200-mesh sieve.

### Starch Granule Size

The analysis of starch granule size was conducted using a laser particle size analyzer (Mastersizer 2000, Malvern, England) as described elsewhere^34^, with minor modifications. Granule sizing experiments were carried out through laser diffractometry using a Mastersizer 2000 instrument, equipped with HydroMu dispersing unit (Malvern). Measurements were taken under the following conditions: anhydrous alcohol mobile phase, particle refractive index of 0.54, particle absorption coefficient of 4, anhydrous alcohol refractive index of 1.366, and a general calculation model for irregular particles. Ten measurement cycles of 10 s each were taken, and the data obtained were averaged using the equipment software (Mastersizer 2000, ver. 5.20 from Malvern).

### Granule Morphology

Granule morphology was examined using the method described by Lu *et al*.^35^, with minor modifications. Starch granules that were steeped in 95% ethanol for 4 h were mounted on circular aluminum stubs and air-dried. Subsequently, these were coated with gold, examined under a scanning electron microscope (SEM, XL-30 ESEM, Philips, Netherlands), and then photographed at an accelerating potential of 20 kV.

### Grain Yield and Other Components

The harvested plot size was 22 m^2^ (four 11-m rows at the center of each plot). Mean grain yield and other components were estimated for each treatment at each location.

Statistical Analysis. Analysis of variance (ANOVA) using a linear model was performed using the software Statistical Product and Service Solutions (SPSS Inc., Chicago, IL, USA) to detect the main effects of hybrids, nitrogen, years and their interaction. Treatments were compared by Duncan’s test and differences were declared as statistically significant if *P* < 0.05. Pearson’s correlations were calculated to determine the relationship between different parameters of maize. All graphics were drawn using Sigmaplot 12.5. The results from 2010 and 2012 followed the same trend. So, all of the calculations in the present study based on the averages of 2010 and 2012.
