# Author Correction: Grain and starch granule morphology in superior and inferior kernels of maize in response to nitrogen

**DOI:** 10.1038/s41598-018-25859-x

**Published:** 2018-05-14

**Authors:** Fucheng Zhao, Liquan Jing, Decheng Wang, Fei Bao, Weiping Lu, Guiyue Wang

**Affiliations:** 1 Dongyang Institute of Maize Research, Zhejiang Academy of Agricultural Sciences, Dongyang, Zhejiang 322100 China; 2 Key Laboratory of Crop Genetics and Physiology of Jiangsu Province, Yangzhou University, Yangzhou, Jiangsu 225009 China; 3 Jiangsu Hongqi Seed Co., Ltd., Taizhou, Jiangsu 225300 China

Correction to: *Scientific Reports* 10.1038/s41598-018-23977-0, published online 20 April 2018

In the original version of this Article, Guiyue Wang was incorrectly affiliated with ‘Jiangsu Hongqi Seed Co., Ltd., Taizhou, Jiangsu, 225300, China’. The correct affiliation is listed below.

Dongyang Institute of Maize Research, Zhejiang Academy of Agricultural Sciences, Dongyang, Zhejiang, 322100, China.

This error has now been corrected in the PDF and HTML versions of the Article.

